# Selection for Heterozygosity Gives Hope to a Wild Population of Inbred Wolves

**DOI:** 10.1371/journal.pone.0000072

**Published:** 2006-12-20

**Authors:** Staffan Bensch, Henrik Andrén, Bengt Hansson, Hans Chr. Pedersen, Håkan Sand, Douglas Sejberg, Petter Wabakken, Mikael Åkesson, Olof Liberg

**Affiliations:** 1 Department of Animal Ecology, Lund University Lund, Sweden; 2 Grimsö Wildlife Research Station, Department of Conservation Biology, Swedish University of Agricultural Sciences Riddarhyttan, Sweden; 3 Division of Terrestrial Ecology, Norwegian Institute for Nature Research Trondheim, Norway; 4 Hedmark University College, Faculty of Forestry and Wildlife Management Koppang, Norway; University of Canterbury, New Zealand

## Abstract

Recent analyses have questioned the usefulness of heterozygosity estimates as measures of the inbreeding coefficient (*f*), a finding that may have dramatic consequences for the management of endangered populations. We confirm that *f* and heterozygosity is poorly correlated in a wild and highly inbred wolf population. Yet, our data show that for each level of *f*, it was the most heterozygous wolves that established themselves as breeders, a selection process that seems to have decelerated the loss of heterozygosity in the population despite a steady increase of *f*. The markers contributing to the positive relationship between heterozygosity and breeding success were found to be located on different chromosomes, but there was a substantial amount of linkage disequilibrium in the population, indicating that the markers are reflecting heterozygosity over relatively wide genomic regions. Following our results we recommend that management programs of endangered populations include estimates of both *f* and heterozygosity, as they may contribute with complementary information about population viability.

## Introduction

Inbreeding is more likely to take place in small populations and may contribute to further decline and eventual extinction [1], [2]. It has therefore become a key objective for conservation geneticists to monitor genetic variation [3] and to measure the occurrence of inbreeding in threatened populations [4]. In the absence of pedigree data, which is the case for the vast majority of endangered animals and plants, measures of average multilocus heterozygosity (MLH) have commonly been used as a proxy for inbreeding coefficients in order to identify the costs of inbreeding (i.e. inbreeding depression). Recent analyses have questioned the usefulness of MLH estimates as measures of the inbreeding coefficient (*f*) [5]. If this is a general problem, as both simulated [6] and empirical data [7] suggest, it will have dramatic consequences for the interpretations of heterozygosity estimates in conservation of endangered populations. Several studies have documented that MLH may correlate with various fitness traits even in situations when *f* is held constant, suggesting that MLH contributes with complementary information about phenotypic and reproductive deterioration above that revealed by measuring inbreeding from pedigree data [8]–[11]. Unfortunately, very few studies to date have simultaneously investigated *f* and MLH and information is therefore missing to what extent these variables may have separate effects on fitness [11].

**Figure 1:**
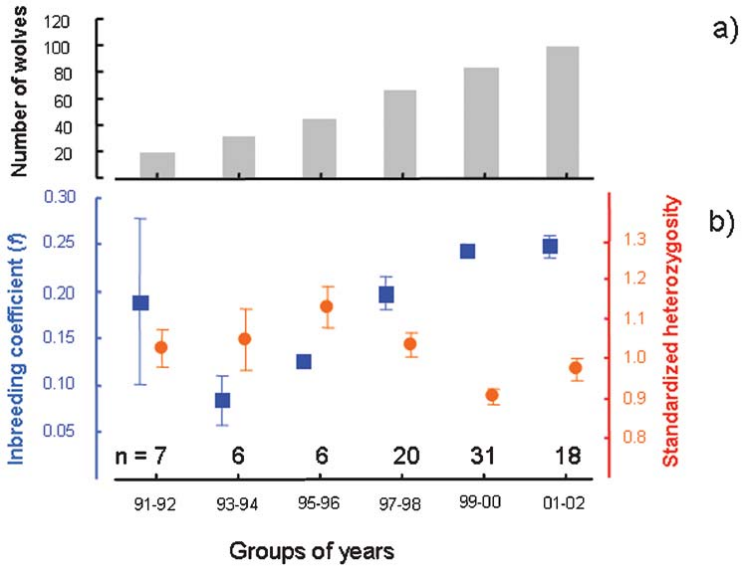
Demographic and genetic data for the Scandinavian wolf population between 1991 and 2002 averaged over two-year periods. a) mean number of wolves and b) mean (±s.e.m.) inbreeding coefficients (blue) and standardized heterozygosity (red). The number of genotyped wolves (*n*) per group of years is indicted above the X-axis. Both inbreeding coefficients (*F*_5,82_ = 5.95, *P*<0.001) and standardized heterozygosity (*F*_5,82_ = 4.97, *P*<0.001) show significant differences among the groups of years.

In this study, we examined the associations between breeding success, heterozygosity at 31 microsatellite loci and pedigree based inbreeding coefficients in a population of wild wolves *Canis lupus* in Scandinavia. This population is highly inbred and has previously been shown to suffer from inbreeding depression as manifested by a reduction in the number of surviving pups during the first winter in inbred litters [12] and an overall higher incidence of vertebral malformations [13]. It presently consists of about 135–152 individuals [14], all stemming from three founding individuals (two started breeding in 1983 and one in 1991) that likely originated from the much larger Finnish-Russian population [15], [16]. Until 1991, when the third immigrant was established in the population, there was only one reproducing pack of wolves, resulting in strong inbreeding and loss of heterozygosity [12], [16]. Following the establishment of this new wolf male, the population heterozygosity increased as did both the number of wolves and breeding packs (Figure 1A), suggesting the importance of this immigrant individual to the successful expansion of the species in Scandinavia [16].

As a measure of fitness in the present study, we used data on whether individual wolves have successfully recruited as breeders into the population and test the hypothesis that the inbreeding coefficient (*f*) is a better predictor of fitness than is multilocus heterozygosity (MLH). The expected strengths of the associations between these variables and fitness should depend on factors such as the intensity of selection for heterozygosity, the level of linkage disequilibrium in the population and the action of genetic drift [17], [18]. To address these points we first mapped the genetic markers to the chromosomes in the dog genome and calculated the level of linkage disequilibrium of the markers in the population. Within cohorts of wolves, we then estimated the selection intensity for MLH and compared the observed level of selection with two independent estimates of the effective population size (N_e_), the parameter determining the predicted magnitude of genetic drift [19]. Our results confirm that *f* and MLH are poorly correlated, and it appears that MLH is strongly associated to our fitness measure independent of the effect of *f*. Over generations, the observed intensity of selection was substantially higher than the potential for genetic drift to change allele frequencies. Selection promoting heterozygotes therefore seems a likely explanation also to why the population largely has maintained the level of heterozygosity despite increased inbreeding coefficients across years.

## Results

The inbreeding coefficients (*f*) in cohorts of wolves that incidentally dropped as a result of the immigrant male in 1991, has again started to accumulate (Figure 1B) and approached *f* = 0.25 in the cohorts born in 2001 and 2002, a level corresponding to full-sib mating. Unexpectedly, standardized mean heterozygosity (stMLH) at 31 microsatellite loci decreased only slightly (Figure 1B).

**Figure 2:**
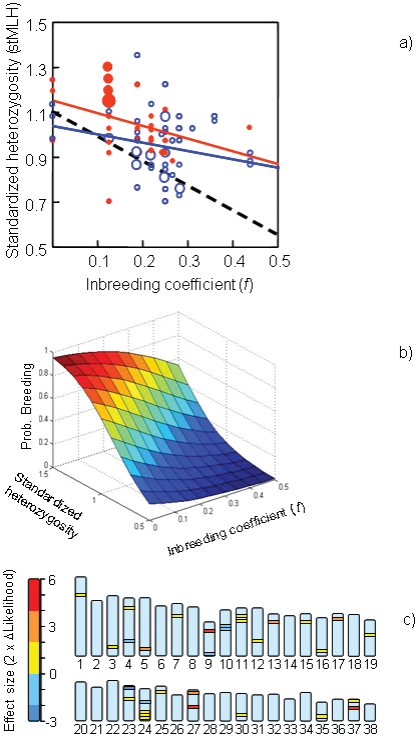
Reproductive status and inbreeding coefficients of Scandinavian wolves in relation to heterozygosity and chromosomal location of 31 microsatellite loci. a) The relationship between inbreeding coefficient (*f*) and standardized heterozygosity (stMLH). Individuals that recruited to the breeding population (filled red circles, solid red regression line) exhibited higher stMLH than those that did not enter the breeding population (open blue circles, solid blue regression line) (ANCOVA: inbreeding coefficient, *F*_1,82_ = 7.96, *P* = 0.006; breeding recruitment success, *F*_1,82_ = 7.43, *p* = 0.008). The stippled black line shows the expected relationship between *f* and stMLH. b) Breeding probability against inbreeding coefficient (*f*) and stMLH based on model estimates from a logistic regression analysis (*f*, β = −5.84, *p* = 0.06; stMLH, β = 4.87, *p* = 0.017). Relative to the population mean values of *f* (0.207) and stMLH (1.0), an increase of 1 SD in *f* corresponds to a 32% reduction in breeding probability, and a decrease of 1 SD in stMLH corresponds to a 40% reduction in breeding probability c) The effects of heterozygosity on the recruitment success of wolves for each of the 31 microsatellite markers and their locations on the autosomal chromosomes in the dog genome. The statistical effect is measured as two-times the likelihood difference between the model with the marker and the null model; positive values (yellow-red) indicate positive associations, negative values (blue) negative associations.

The two indices of individual genetic variation, *f* and stMLH, were negatively correlated (*r* observed = −0.388, Figure 2A), however not as strongly as expected (*r* expected = −0.532) following equation 4 in Slate et al. [7]. The expected correlation was calculated using the following parameters estimated from the data as input; mean [(*f*) = 0.209] and variance [σ^2^(*f*) = 0.009] of the inbreeding coefficient, mean number of scored loci [(L) = 30.4] and mean heterozygosity (MLH = 0.596).

A way of testing whether the observed relationship between *f* and stMLH is weaker than the expected relationship is to compare the observed regression slope with the predicted slope assuming that MLH is 0 when *f* = 1. By assuming an average stMLH of 1.10 for non-inbred wolves, taken from the mean of the six individuals with *f* = 0, we found that the slope (*b* observed = −0.594±0.155) was significantly more shallow than the predicted slope (*b* predicted = −1.10) between stMLH and inbreeding coefficients (Figure. 2A; *t* = 3.26, d.f. = 83, *p*<0.01). This result suggests that for each level of *f* we find fewer homozygous wolves than expected. To examine whether this disparity is due to selection against homozygous individuals up to the event of sampling, we compared the observed MLH (mean 0.593) for each individual with the expected MLH (mean 0.579) as calculated from its parental genotypes. However, the observed and expected MLH were not significantly different (*t* = 1.29, d.f. = 38, *p* = 0.2, paired t-test) suggesting that heterozygosity of offspring is no different from what is expected under Mendelian inheritance.

Instead the explanation to why we detect less homozygous wolves than expected seems to be found in a difference between breeders and non-breeders. The level of stMLH was significantly higher for wolves that established as breeders (*n* = 32) compared to those never entering the breeding population (*n* = 53) (ANCOVA, *p* = 0.007; controlling for *f*; Figure 2A). That stMLH is in fact a stronger predictor of breeding probability than is *f* is supported by a multiple logistic regression analysis (Figure 2B).

**Figure 3:**
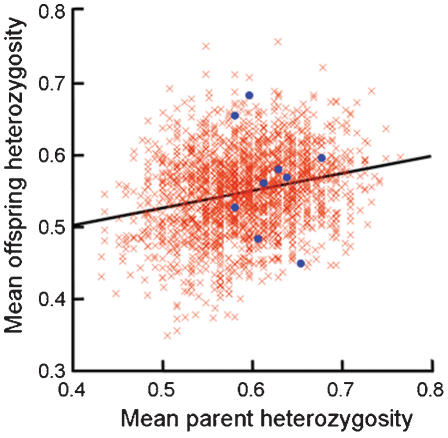
Simulated mean heterozygosity of 100.000 offspring from 2068 pairs of wolves regressed on the mean heterozygosity of the parents. The filled blue circles represent the simulated heterozygosity of offspring from nine actual pairs in the Scandinavian wolf population. The regression slope is 0.241 and r^2^ is 0.057.

It is important to understand how genetic variation can be maintained in populations accumulating inbreeding coefficients. We therefore investigated whether heterozygosity was heritable, but there is no evidence for this (midparent-midoffspring regression r = −0.37, *n* = 9, *p* = 0.27) though our sample size is small due to lack of full genotypes from several breeding individuals. However, the important question to ask is whether the offspring from the relatively more heterozygote wolves that did reproduce, were more heterozygote than offspring that could have been produced from wolves picked randomly from the population. We studied this by a simulation approach, where haplotypes from randomly drawn pairs of one male and one female from the population were combined into 100,000 offspring genotypes. The mean MLH of the simulated offspring from the nine “real pairs” in the population was 0.567 (±0.100 S.D.) and it was only slightly higher than the mean of 0.548 (±0.094 S.D.) of all simulated offspring. When regressing the mean MLH of the offspring from each parental pair (n = 2068) on the mean MLH in parents we found a regression coefficient of 0.241 (r^2^ = 0.057), which suggests that more heterozygous parents are more likely to produce more heterozygous offspring (Figure 3). It is also noteworthy that more heterozygous parent pairs had offspring that varied more in MLH than parents with low heterozygosity (regression of S.D. of offspring MLH on mean parent MLH; b = 0.35)

The level of inbreeding depression in the Scandinavian wolf population was estimated by measuring “breeding-failure equivalents” in the genome (analogue to lethal equivalents) and was found to be 5.42 (95% CI: 1.10–12.26). To examine whether the observed effects of heterozygosity on recruitment success can be explained by a few loci with strong effects, or many loci with small effects, we used logistic regression analyses to calculate the association between heterozygosity and recruitment success for each locus separately. For the majority of loci (25 of 31), heterozygosity was positively associated with recruitment success, although only three of these associations were significant (AHT002, *p* = 0.02; AHT133, *p* = 0.045; 250, *p* = 0.05), all on different chromosomes (Figure 2C).

**Figure 4:**
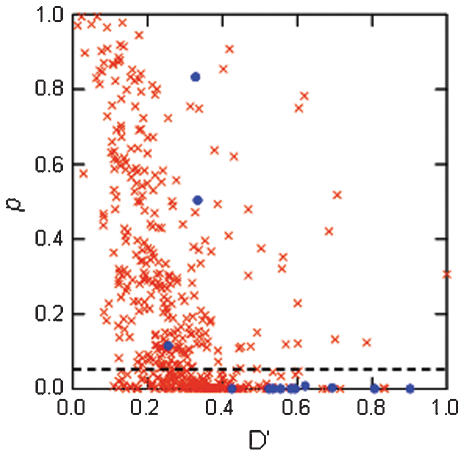
Linkage disequilibrium between pair-wise microsatellite loci in Scandinavian wolves. D′- and *p*-values are shown for locus-pairs located on the same (dots) and different (crosses) chromosomes. The dashed line indicates the *p* = 0.05 significance level.

There was strong linkage disequilibrium (LD) within chromosomes: 10 of 13 locus-pairs (77%) located on the same chromosome had significant LD (*p*<0.05) with an average D′ of 0.624 (range: 0.43–0.90; Figure 4). As expected, LD was less pronounced for loci located on different chromosomes: 168 of these 454 locus-pairs (37%) had significant LD (*p*<0.05) with an average D′ of 0.344 (range: 0.11–0.83; Figure 4).

Selection differentials (*S*) and selection intensities (*i*), i.e. the standardized selection differentials, were calculated for all cohorts of wolves containing both breeders and non-breeders (Table 1). In the following we only refer to the selection intensities (*i*) as this is the standardised parameter that can be directly compared with the effect of genetic drift. The selection intensity (*i*) was positive in six of the seven cohorts and on average tended to be different from zero (*t* = 2.01, *p* = 0.09). For the whole period of the eleven cohorts, it was estimated to be 0.491 (*t* = 3.76, df = 86, *p*<0.001; two-sample t-test). Note that the total selection intensity for the whole period does not correspond to the average values for the cohorts.

**Table 1:**
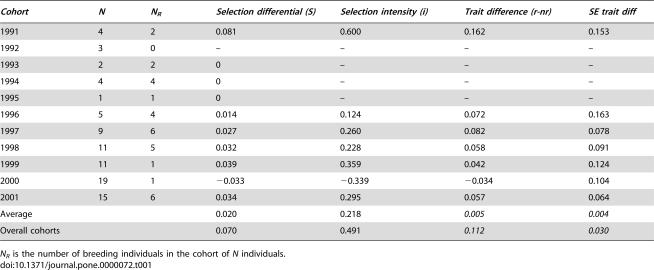
Selection differential (S) and selection intensity (*i*) on standardized heterozygosity and trait differences between wolves recruited (r) and not recruited (nr) to the breeding population.

*Cohort*	*N*	*N_R_*	*Selection differential (S)*	*Selection intensity (i)*	*Trait difference (r-nr)*	*SE trait diff*
1991	4	2	0.081	0.600	0.162	0.153
1992	3	0	–	–	–	–
1993	2	2	0	–	–	–
1994	4	4	0	–	–	–
1995	1	1	0	–	–	–
1996	5	4	0.014	0.124	0.072	0.163
1997	9	6	0.027	0.260	0.082	0.078
1998	11	5	0.032	0.228	0.058	0.091
1999	11	1	0.039	0.359	0.042	0.124
2000	19	1	−0.033	−0.339	−0.034	0.104
2001	15	6	0.034	0.295	0.057	0.064
Average			0.020	0.218	*0.005*	*0.004*
Overall cohorts			0.070	0.491	*0.112*	*0.030*

*N_R_* is the number of breeding individuals in the cohort of *N* individuals.

In order to compare the observed selection intensity with the effect of genetic drift we used two different estimates of the effective population size. From demographic data and using the program VORTEX [19], *N*_e_ for the current population (135–152 wolves) was previously estimate to be around 47–53 individuals [20], however these calculations are complicated due overlapping generations and that the population has increased during the study period. An independent estimate of the effective population size was obtained by analysing variation in microsatellite allele frequencies between cohorts in the program NeEstimator [21], and with this method we found a similar *N*_e_ (45.6; 95% CI: 20.4–181.2). Assuming this *N*_e_, the identified per generation selection intensity (0.245) for heterozygosity is 22 times higher than the effect of genetic drift (1/(2*45.6) = 0.011).

## Discussion

Though still globally distributed in the northern hemisphere, the wolf has in some parts of the world declined to only a fraction of its historical numbers, recently confirmed by genetic analyses of populations in both North America [22] and Europe [23], [24]. One may argue that the Scandinavian wolf population is extreme in its level of inbreeding, as it was founded by only three individuals [12], [16] and now having an average inbreeding coefficient of 0.25, corresponding to the mating between full sibs. However, similar degree of isolation and small population-sizes are sadly common features of many large carnivore species. We therefore trust that the patterns outlined here will help to better understand the genetics of small and endangered populations of other species.

In this study we found that for each level of inbreeding, it was the most heterozygous wolves that were recruited into the breeding population, i.e. we found evidence for selection against homozygous individuals. We argue that this selective process can explain both why the population is not losing stMLH at the same rate as *f* is increasing (Figure 1B) and the unexpectedly poor correlation between these variables (Figure 2A). Although we found statistical evidence for this correlation to be weaker than expected, we cannot fully rule out that errors in the pedigree might have introduced errors in *f* and thus weakened its correlation with stMLH. However, we feel confident that we have data from all but one reproducing pair since 1991, and since all individual genotypes have been compared with all possible parent-combinations in the population, the number of errors in the pedigree should be small [12]. Also, the most likely error would be to incorrectly assign parentage to a brother or sister of the reproducing male or female, respectively, however such a mistake would not affect the estimate of *f*. We therefore trust that the weak correlation between *f* and stMLH indeed is not resulting from errors in the pedigree. In contrast to our results, a previous study of Scandinavian wolves found good agreement between MLH and *f* [25]. However, that study was done on wolves in captivity so the effects of natural selection and mate choice may have been reduced. This agrees with previous findings demonstrating that cost of inbreeding is context dependent [26] and stronger in natural environment than in captivity [27].

The cost of inbreeding in the Scandinavian wolf population seems substantial compared to other species of mammals [28], [29]. The estimate obtained here of “breeding-failure equivalents” of 5.42 is similar to the “litter-reducing equivalents” of 6.04 in this population that we previously obtained from a study of inbreeding depression of number of pups recruited per litter into their first winter [12]. Because heterozygosity has not dropped in parallel with the inbreeding coefficient (Figure 1B) and selection appears to act to maintain heterozygosity (Figure 2B), the calculated figure of breeding failure equivalents is probably an underestimate of the true number of detrimental alleles. The number detrimental alleles in a population founded by few individuals, probably depends heavily on the particular founders since the number and composition of detrimental alleles will differ between individuals in the source population [30]. Hence, a different set of three founding wolves from the Finnish-Russian population may have resulted in a lower cost of inbreeding. In turn, this would have reduced the advantage for heterozygotes and lowered the selection intensity, generating a more concordant change of *f* and stMLH as well as strengthening the correlation between the variables.

We found that the loci contributing to the correlation between stMLH and breeding success mapped to different chromosomes and that the level of linkage disequilibrium was high within chromosomes. These results suggest that the detected association between stMLH and probability of breeding is driven by several loci with smaller effects and that our microsatellite-based measure of heterozygosity reflects heterozygosity over a substantial part of the genome. The variation in genome-wide heterozygosity that selection acts upon in this wolf population might have been caused in two ways. First, the pronounced variation in *f* in the population (Figure 2A) causes variation in genome-wide heterozygosity. Second, when there is strong linkage disequilibrium (LD) in a population there are relatively few segregating chromosome units and then random segregation will have a marked influence on the variation in genome-wide heterozygosity also within each level of inbreeding [11]. Moreover, of importance for our correlation between measured heterozygosity and probability of breeding is also that random segregation induces variation in heterozygosity in the local chromosomal vicinity of the markers, i.e. the parts of the chromosomes in LD with the markers. When there is much LD, selection on variation in heterozygosity on fitness loci located in these local but rather extensive regions of the genome can result in correlations between measured heterozygosity and variation in fitness-associated traits also within each level of inbreeding (B. Hansson and L. Westerberg, in prep.). Our analyses confirmed that the level of LD in the Scandinavian wolf population is substantial, with significant D′-values ranging between 0.42 and 0.90 within chromosomes. This level of LD is considerably higher than found in many other natural populations [31], [32], and more similar to the high levels detected among some domesticated and artificially selected populations [33], [34]. It is probable that the high LD in the studied wolf population has been caused by the strong bottleneck and recent expansion with ongoing inbreeding.

In populations having small effective population sizes (*N*_e_) genetic drift is a strong force often over-riding the effect of selection (*S*) and this happens when 1/2*N*_e_>*S* [17]. Summed over the studied cohorts, we found the selection intensity *i* for heterozygosity to be 0.49 (Table 1). Assuming a generation time of 5.5 years as previously estimated for this wolf population [20], the per generation selection intensity is 0.245, which operationally would require an effective population size above two individuals only. Hence, the strength of selection identified in our population could potentially work also in the smallest of populations.

At present, we cannot distinguish whether the higher success of heterozygous wolves to recruit to the breeding population is caused by selection on survival to breeding age or factors determining pair formation and successful mating. Irrespective of the mechanism, selection is a likely explanation to the population maintaining relatively high levels of heterozygosity despite accumulating levels of inbreeding. We identify three circumstances that may have facilitated the maintenance of heterozygosity in this population. First, the population has increased five-fold during the study period (Figure 1A), allowing more power for selection than if the population had been constant or declining [35]. Second, the population was founded by three presumably unrelated and outbred individuals as recently as zero to three generations before this study was commenced [12]. Third, we find from a simulation analysis that the average heterozygosity in offspring are positively correlated by the average heterozygosity of the parents. This suggests that the population has the potential to show “response” to the selection on heterozygosity. However, we find from our simulation only weak (or no) indications that the pairs that actually did reproduce would get offspring with higher expected heterozygosity than if mating was random. The low number of recombination events certainly contributes to the observed high level of LD, which enables selection on heterozygosity over relatively wide genomic regions. Our study demonstrates that small isolated populations in the wild may not lose genetic variation as quickly as predicted from neutral population genetic theory. This is particularly likely to apply to new populations founded by a few individuals or small populations recruiting immigrants.

## Materials and Methods

### Field data and genetic analyses

The Scandinavian wolf population has been monitored since 1978, based on snow tracking and, from 1998, also on radio telemetry. Details for determining number of wolves, identification of breeding units, and criteria for determining successful breeding, are given in Wabakken et al. [36]. Samples for microsatellite analyses were derived from blood of captured wolves, muscle of dead wolves, from oestrus blood on snow and from scats, and were analysed as previously described [12]. Scat samples were amplified four to ten times per locus to circumvent inferring non-complete genotypes [37]. Individuals from which genotypic data were available from scats only, were used for reconstructing the pedigree [12] but were excluded from the here presented analyses of individual heterozygosity as genotyping errors cannot fully be circumvented when using low quality DNA. The details of microsatellite primers are given in Table S1 and the procedure of the reconstruction of the pedigree in the electronic appendix to Liberg et al. [12]. As a measure of fitness in the present study, we used data on whether individual wolves sampled during their first (*n* = 7), second (*n* = 36), third (*n* = 16) or later calendar years (*n* = 28), have successfully recruited as breeders into the population. This variable combines survival until reproductively mature, which is about two years for both sexes in the population (unpubl.), and the success of becoming an established breeding individual.

Genome locations of microsatellite loci (see Table S1) were determined by running BLAST analyses for the two primer sequences of each locus on the *Canis familiaris* genome (http://www.ensembl.org/Canis_familiaris/index.html; BLAST settings: ‘near-exact matches’ with W = 8). In all cases a single highly significant location (Figure 2C) was detected and the length of the matching region was similar to the length of the amplified wolf PCR products.

### Statistical analyses

Multilocus heterozygosity (MLH) is calculated as the proportion of heterozygote loci among loci typed (varied between 27 and 31). Standardized heterozygosity (stMLH) is calculated as MLH divided by the population mean MLH for these loci. In the statistical analyses we used stMLH rather than MLH as it adjusts for the variation in the analysed loci, however the two measures are strongly correlated (*r* = 0.998) and all conclusions remain unchanged when the analyses are based on MLH. For the analyses of the effect of heterozygosity and inbreeding coefficients we included all wolves that were typed at 27–31 microsatellite loci as previously described [12] and had estimated birth years between 1991 and 2001 (*n* = 87). This excludes the three founding and supposedly outbred individuals (*f* = 0) that entered as breeders in the population in 1983 (two) and 1991 (one), respectively. Also, the majority of individuals being recruited into the breeding populations are 2–4 years, thus allowing the 2001 cohort to be evaluated for reproduction status without bias. We were not able to place two of the individuals in the pedigree due to lack of DNA samples from one breeding territory (territory Xa3 in Liberg et al. [12]) and these were therefore excluded as we did not have accurate estimates of their inbreeding coefficients. The total data set consisted therefore of 85 individuals. For the analyses of average heterozygosity and inbreeding coefficients per groups of cohorts (Figure 1) we also included data from three individuals from 2002.

Inbreeding depression is estimated using a method developed by Morton et al. [38] and improved by Kalinowski and Hedrick [39]. We calculated breeding-failure equivalents in an analogous fashion to the calculation of lethal equivalents with maximum-likelihood [39] to overcome the problem of zero breeding success at one or more breeding levels. Because of small sample sizes within years we pooled all years in the analysis.

We estimated the directional selection differential [40] *S* and selection intensity [40] *i* for standardized heterozygosity (stMLH) as the difference between means of the characters before and after selection [41]. The selection intensity *i* is the standardized selection differential with stMLH standardized to a mean of zero and a variance of one [42]. Individuals after selection consists of those that successfully managed to reproduce later in life. Selection differentials were calculated for each cohort separately and overall cohorts. Significance of *S* and *i* was tested by comparing stMLH and standardized stMLH of breeders and non-breeders using two-sample t-tests [43].

Significance tests of linkage disequilibrium between all pairs of loci were performed in the program Arlequin (Ver. 2; http://cmpg.unibe.ch/software/arlequin/) using 10,000 permutations. Pair-wise D′-values were calculated using a function written in R (K. Csilléry, in prep.). Haplotype frequencies were inferred from the genotype data with the EM algorithm implemented in the ‘haplo.stats’ library of R. Effective population size was estimated from allele frequency data for the 31 microsatellites with the program NeEstimator (Ver. 1.3) [21] using six temporal classes (corresponding to those in Figure 1A).

We simulated in Excel whether multilocus heterozygosity is likely to be heritable in our population. This was done by generating 100,000 offspring genotypes from parental pair combination drawn randomly from the 44 females and 47 males from which we had complete genotypes (i.e. 2068 possible pairs). Upon every simulated pairing, a haplotype from each parent was generated by random selection of alleles from their microsatellite genotypes. These haplotypes were combined to form an offspring genotype. The multilocus heterozygosity was calculated for the offspring and analyzed in relation to the parent heterozygosity in SPSS version 14.0.

## Supporting Information

Table S1 Number of alleles and expected and observed heterozygosity in the contemporary Scandinavian wolf population for 31 microsatellite loci used in the present study.
(0.06 MB DOC)
