# Supplementary material for: Selection for Heterozygosity Gives Hope to a Wild Population of Inbred Wolves
Source: PLoS One. 2006 Dec 20;1(1):e72. doi: 10.1371/journal.pone.0000072 (PMC1762340; doi:10.1371/journal.pone.0000072)
Supplement: Table S1 — Number of alleles and expected and observed heterozygosity in the contemporary Scandinavian wolf population for 31 microsatellite loci used in the present study. (0.06 MB DOC) [file pone.0000072.s001.doc]

Supplementary Table 1. Number of alleles and expected and observed heterozygosity in the contemporary Scandinavian wolf population for 31 microsatellite loci used in the present study. Location of the loci in the dog genome and references to primer publications are also given.

| Locus | No. alleles | Exp. het. | Obs. het. | Chromosome (position) | Reference* |
| --- | --- | --- | --- | --- | --- |
| CXX.20 | 3 | 0.63 | 0.69 | 11 (63685297) | [S1] |
| CXX.109 | 2 | 0.35 | 0.38 | 4 (23538070) | [S1] |
| CXX.123 | 3 | 0.54 | 0.63 | 23 (31791232) | [S1] |
| CXX:173 | 3 | 0.36 | 0.42 | 9 (3915363) | [S1] |
| CXX.204 | 3 | 0.65 | 0.73 | 30 (8047991) | [S1] |
| CXX.213 | 4 | 0.63 | 0.68 | 25 (44075755) | [S1] |
| CXX.225 | 3 | 0.50 | 0.53 | 10 (43403385) | [S1] |
| CXX.250 | 4 | 0.68 | 0.70 | 9 (37477620) | [S1] |
| 2001 | 5 | 0.37 | 0.37 | 23 (50965337) | [S2] |
| 2010 | 3 | 0.66 | 0.69 | 24 (5205899) | [S2] |
| 2054 | 3 | 0.66 | 0.68 | 12 (24218131) | [S2] |
| 2088 | 4 | 0.66 | 0.63 | 15 (53916786) | [S2] |
| 2137 | 5 | 0.79 | 0.84 | 3 (13990289) | [S2] |
| 2140 | 4 | 0.50 | 0.56 | 5 (12643505) | [S2] |
| 2159 | 4 | 0.34 | 0.24 | 24 (12659622) | [S2] |
| 2168 | 4 | 0.60 | 0.66 | 24 (11096784) | [S2] |
| 2201 | 4 | 0.67 | 0.72 | 7 (62338840) | [S2] |
| (AHT)002 | 4 | 0.73 | 0.63 | 27 (21738669) | [S3] |
| (AHT)004 | 3 | 0.55 | 0.64 | 37 (31321434) | [S3] |
| (AHT)101 | 4 | 0.54 | 0.55 | 10 (46440833) | [S3] |
| (AHT)106 | 2 | 0.50 | 0.58 | 16 (8120998) | [S3] |
| vWF | 6 | 0.74 | 0.76 | 27 (41929532) | [S4] |
| AHT126 | 3 | 0.41 | 0.42 | 17 (58212221) | [S5] |
| AHT124 | 2 | 0.49 | 0.52 | 19 (33142278) | [S5] |
| AHT133 | 3 | 0.07 | 0.06 | 37 (18055969) | [S6] |
| AHT138 | 4 | 0.55 | 0.62 | 1 (96976083) | [S6] |
| AHT119 | 3 | 0.45 | 0.48 | 35 (6770648) | [S6] |
| AHT121 | 5 | 0.78 | 0.84 | 13 (51810088) | [S6] |
| AHT136 | 2 | 0.48 | 0.49 | 11 (54459932) | [S6] |
| AHT103 | 4 | 0.54 | 0.61 | 4 (75313498) | [S6] |
| AHT125 | 3 | 0.59 | 0.64 | 24 (29817831) | [S5] |
|  |  |  |  |  |  |

[S1] Ostrander EA., Sprague GF, Rine J. (1993) Identification and characterization of dinucleotide repeat (ca)n markers for genetic-mapping in dog. *Genomics* **16**, 207 – 213.

[S2] Fransisco LV, Langston AA, Mellersh CS, Neal CL, Ostrander EA (1996) A class of highly polymorphic tetranucleotide repeats for canine genetic mapping. *Mammalian Genome* **7,** 359 – 362.

[S3] Holmes NG, Mellersh CS, Humphreys SJ, Binns MM, Holliman A, et al. (1993) Isolation and characterization of microsattelites from the canine genome. *Animal Genetics* **24**, 289 – 292.

[S4] Shibuya H, Collins BK, Huang TH, Johnson GS (1994) A polymorphic (AGGAAT)n tandem repeat in an intron of the canine von Willebrand factor gene. *Animal Genetics* **25**, 122.

[S5] Holmes NG, Strange NJ, Binns MM, Mellersh CS, Sampson J (1994) Three polymorphic canine microsatellites. *Animal Genetics* **25**, 200

[S6] Holmes NG, Dickens HF, Parker HL, Binns MM, Mellersh CS, et al. (1995) Eighteen canine microsatellites. *Animal Genetics* **25**, 132 – 133.
